# Insight, psychopathology, explanatory models and outcome of schizophrenia in India: a prospective 5-year cohort study

**DOI:** 10.1186/1471-244X-12-159

**Published:** 2012-09-27

**Authors:** Shanthi Johnson, Manoranjitham Sathyaseelan, Helen Charles, Visalakshi Jeyaseelan, Kuruthukulangara Sebastian Jacob

**Affiliations:** 1 College of Nursing, Christian Medical College, Vellore, 632004, India; 2 Christian Medical College, Vellore, 632002, India

**Keywords:** Schizophrenia, Outcome, Insight, Explanatory models, Culture

## Abstract

**Background:**

The sole focus of models of insight on bio-medical perspectives to the complete exclusion of local, non-medical and cultural constructs mandates review. This study attempted to investigate the impact of insight, psychopathology, explanatory models of illness on outcome of first episode schizophrenia.

**Method:**

Patients diagnosed to have DSM IV schizophrenia (n = 131) were assessed prospectively for insight, psychopathology, explanatory models of illness at baseline, 6, 12 and 60 months using standard instruments. Multiple linear and logistic regression and generalized estimating equations (GEE) were employed to assess predictors of outcome.

**Results:**

We could follow up 95 (72.5%) patients. Sixty-five of these patients (68.4%) achieved remission. There was a negative relationship between psychosis rating and insight scores. Urban residence, fluctuating course of the initial illness, and improvement in global functioning at 6 months and lower psychosis rating at 12 months were significantly related to remission at 5 years. Insight scores, number of non-medical explanatory models and individual explanatory models held during the later course of the illness were significantly associated with outcome. Analysis of longitudinal data using GEE showed that women, rural residence, insight scores and number of non-medical explanatory models of illness held were significantly associated with BPRS scores during the study period.

**Conclusions:**

Insight, the disease model and the number of non-medical model positively correlated with improvement in psychosis arguing for a complex interaction between the culture, context and illness variables. These finding argue that insight and explanatory models are secondary to psychopathology, course and outcome of the illness. The awareness of mental illness is a narrative act in which people make personal sense of the many challenges they face. The course and outcome of the illness, cultural context, acceptable cultural explanations and the prevalent social stigma interact to produce a complex and multifaceted understanding of the issues. This complexity calls for a nuanced framing of insight.

## Background

The term insight, employed in the context of self-awareness, has been used in many different ways. It ranges from a basic awareness of one’s situation to a deeper intellectual understanding and emotional appreciation of issues. It has also been used differently by different disciplines (e.g. psychoanalysis) to imply particular understanding. Assessment in clinical psychiatry terms insight as “a patient’s capacity to understand the nature, significance and severity of his or her illness” [1]. Such awareness has major clinical implications for phenomenology, clinical management, coping, help seeking and treatment compliance.

Research related to insight and schizophrenia has changed substantially over the past few decades. Older trans-cultural studies by the World Health Organization employed uni-dimensional and all-or-none perspectives in the elicitation of insight [2,3]. They concluded that the majority of people with schizophrenia “lacked insight”. This was consistently demonstrated in acute and chronic schizophrenia and across national and cultural settings. Recent investigations have employed a more sophisticated approach to insight and employed multi-dimensional perspectives [4-9]. These models include the awareness of one’s suffering, the appreciation of certain beliefs, perceptions and experiences and their medical implications [4,5]. The concepts and instruments focus on relabeling of the experience, recognition of mental illness and seeking medical and psychiatric help. These assessment have been used to study the correlation between insight, psychopathology, explanatory models, disability, social issues and biology [6,8-13].

Nevertheless, the more recent approaches have also been criticised [14-17]. Their sole focus on the biomedical/disease model, the cost of labeling and stigma have been highlighted as limitation. Investigators have highlighted the arrogance of bio-medical views as local culture and non-western beliefs are excluded even when such investigations are done in the non-western world.

Many investigations, from non-western cultures, have recorded the simultaneous presence of multiple and contradictory models of illness in people [8,18-23]. Non-medical beliefs (e.g. karma, evil spirits, black magic, sin, punishment by God, etc.) compete with biomedical concepts of illness (e.g. disease, degeneration, deficiency, etc.). Patients select the health care system from the diverse range of facilities available for cure and healing. The co-existence of traditional and modern systems of medicine means that these facilities compete for providing health care to people with different illnesses. This is considered the norm in low and middle income countries but has also been recorded in western populations [20,24].

Pluralistic societies employ multiple approaches to health and illness. Disease models of causation are almost universal in rural India and in low and middle-income countries for illnesses of short duration (e.g. fever, diarrhea [25,26]). In fact, physicians are under pressure to provide immediate relief from symptoms. On the other hand, people employ multiple explanatory models of illness to explain chronic diseases, especially those with variable response to medical treatment, course and outcome [27]. The simultaneous use of contradictory models suggests their use in coping with different aspects of the condition.

This study attempted to study the impact of local cultural explanations, attributions and actions on the long-term outcome of first episode schizophrenia in Vellore, India. It employs a cohort design where the patients were followed-up for 5 years after the initial recruitment.

## Method

### Study site

This study was carried out in the Department of Psychiatry, Christian Medical College, Vellore, Tamil Nadu, south India. The total area of Vellore district is 4314.29 km^2^. The 122-bed hospital provides short-term care psychiatric care for the town, the district and a much wider rural area beyond. The department treats a variety of mental and behavioural disorders with a multi-disciplinary and eclectic approach using standard pharmacological and psychological therapies. The hospital has a daily outpatient clinic in which about 350–400 patients are seen. Patients requiring hospitalization are admitted for a period of 2–6 weeks.

The hospital follows standard protocols for the treatment of patients with schizophrenia. Antipsychotic medication (e.g. risperidone, olanzapine, chlorpromazine) are drugs of choice and employed in adequate doses (e.g. 4–8 mg of risperidone, 15–25 mg of olanzapine and 400–1000 mg of chlorpromazine) and for a period of about 2 years for people with first episode of psychosis. Clozapine is reserved for patients who do not respond to at least 2 antipsychotic drugs given in adequate dose for at least 6–12 weeks. All patients admitted to the hospital also receive psychoeducation, supportive psychotherapy and occupational therapy. Patients with specific problems related to marriage, sex, interpersonal and vocational issues are helped by psychological interventions (e.g. cognitive and behavioural therapy).

### Sample and procedures

The details of the cohort are described in other publications [8,18,19]. The study group consisted of patients with schizophrenia having their first contact with mental health services and living within a 100 km radius of the study site. Patients were carefully screened for a DSM IV diagnosis of schizophrenia [28]. The details of the study were explained and written informed consent was obtained. The following instruments were employed for assessment at baseline:

(i) *Structured Clinical Interview forDSM-III R Patient version(SCID-P*): We used the Structured Clinical Interview for DSM-III R Patient version (SCID-P) to assess psychopathology and confirm diagnosis [29]. It is a structured and comprehensive interview to assess psychopathology and confirm diagnosis. Patients with a primary diagnosis of substance use disorders, mood disorder or organic mental disorders were excluded. This instrument has been employed in many local studies [30,31].

(ii) *Schedule for Assessment ofInsight Expanded (SAI-E):*[32,33]. The schedule comprises of questions to assess three dimensions of insight: awareness, relabeling of symptoms and compliance plus a ‘hypothetical contradiction’ item added to evaluate the person's capacity to consider another's perspective. Each dimension comprises of two or three questions, which are scored on a 3-point scale. The supplementary question is scored from 0 to 4 and this is added to the total score. The instrument also includes items on awareness of change, difficulties resulting from the mental condition and insight into key symptoms. The instrument has been widely employed in western and non-western cultures [34,35].

(iii) *Brief Psychiatric Rating Scale(BPRS):*[36] The scale is commonly employed to assess patients with psychosis. It has a recommended interview schedule, symptom definitions, and specific anchor points for rating symptoms and behaviors. It has self-report items and those rated based on observed behavior and speech. Each item is rated between 1 and 7.

(iv) *Short Explanatory Model Interview(SEMI):*[20,21]. The interview explores *emic* perspectives of illness. It employs open-ended questions and is semi-structured. The subjects are encouraged to talk openly about their attitudes and experience with the aim of eliciting concepts held, and relationship to current situation and culture. Probes are also employed to confirm the concepts mentioned and to explore areas, which the patients did not volunteer. The interview is divided into five sections to cover the subject's background, nature of presenting problem, help seeking behaviour, interaction with physician/healer, and beliefs related to mental illness. The individuals beliefs related to the nature of the presenting problem are examined in detail and include the reason for consulting, name of the problem, perceived causes, consequences, severity and its effects on body, emotion, social network, home life and on work. *Emic* symptoms are elicited by open-ended probing. Help seeking behaviour, especially contact with alternative non-medical sources (e.g. traditional healers) are also examined. The answers to the questions were recorded verbatim. Items were enumerated and the broad facets identified. Item, which occurred frequently, were allocated independent codes. Subsequently, SEMI data was analysed and *emic* items coded dichotomously (not reported/reported). The SEMI has been translated into many different languages and used among people of different cultures. It has also been used to elicit perspectives among Indian populations. [37-39]. The Tamil version [39-41] was used in this study.

(v) *Global Assessment of Functioning(GAF) *[42]*:* This scale was employed to assess overall function.

We used Tamil versions of the instruments. The cohort was followed up at 6 and 12 months as part of the initial study [8,18,19]. The patients continued to receive treatment at the psychiatric hospital. We then attempted to follow up all the 131 patients at 60 months. We assessed patients who regularly attended the hospital. We visited and interviewed all other patients in their homes.

### Outcome assessments

We used the following instruments to assess outcomes at 5 years: (i) Schedule for Assessment of Insight Expanded (SAI-E), [32,33] (ii) the Brief Psychiatric Rating Scale (BPRS), [36] (iii) Short Explanatory Model Interview (SEMI), [20,21]. We also employed the following instrument to assess outcome:

(i) Positive and Negative Syndrome Scale (PANSS), [43]. The 30-item scale is commonly used to assess people with schizophrenia and psychosis. It is comprehensive and has 7 items for positive symptoms, 7 items for negative symptoms and 16 items to assess general psychopathology. Each item has a 7-point severity scale. Its concurrent and predictive validity and sensitivity to change have been established.

(ii) WHO Disability Assessment Schedule (WHODASS II) [44]. The instruments provides a profile of functioning across six activity domains as well as a global disability score. It can be used to identify needs, match patients with interventions, track functioning across time, and measure clinical outcome and treatment effectiveness. The Tamil version has been used locally [45].

(iii) A pro forma to clinical details and data on medication compliance.

We defined remission as being less than mild on all items P1, P2, P3, N1, N4, N6, G5, G9 on PANSS [46] using standard criteria.

### Statistical analysis

We used descriptive statistics to describe continuous variables and frequency distributions for categorical variables. We calculated odds ratios and their 95% confidence intervals. We employed multiple linear and logistic regression for multivariable analysis. We employed Generalized Estimating Equations (GEE) analysis to analyse longitudinal, repeated and correlated data. We used the SPSS version 16 for analysis.

The Institutional Review Board of the Christian Medical College, Vellore, approved the study protocols.

## Results

Over the 1-year recruitment period, 196 patients with schizophrenia attended the Department of Psychiatry, Christian Medical College, Vellore, and 188 met the entry criteria. Of these 37 were excluded because the severity of psychopathology precluded an interview, 14 did not attend and 6 refused consent, yielding a final sample of 131 participants [8,18,19]. The majority of the sample were young adults (mean age 29.5 years; sd 7.2), male (n = 72; 55%), lived in rural areas (n = 105; 80.2%) and were literate (n = 100; 76.3%). The average age of onset of illness was 27.8 years (sd 6.85) with a mean duration of 95.5 weeks (sd 134.2). 16 (12.2%) were voluntary patients while the rest (n = 115; 87.8%) had involuntary status. The mean BPRS and SAI-E scores were 56.7 (sd 5.2) and 4.7 (sd 4.57) respectively. Many patients held specific causal models of illness including black magic (n = 96; 73.3%), evil spirits (n = 23; 17.6%), punishment by God (n = 14; 10.7%), previous deeds (n = 12; 9.2%) hereditary factors (n = 1; 0.8%), disease (n = 17; 13.7%) and psychosocial factors (n = 14; 10.7%). 29 (22%) of patients held multiple causal models while 47 (35.9%) held multiple treatment models.

Of the 131 patients in the cohort, 95 (72.5%) were followed up at 5 years and interviewed. Five patients had died (suicide = 2; natural death = 3) and the remaining 31 were not traceable as they had moved out of the area. There were no statistically significant differences between the subjects lost to follow up and those who participated in the study at 5 years on the following baseline variables: age, sex, residence, literacy, education, employment, family history of mental illness, age of onset of illness, number of non-medical explanatory models, BPRS and SAI-E scores and visits to traditional healers.

### 5-year outcome

Table 1 records details of course and outcome over the 5-year period. The majority of subjects met remission criteria while a minority reached their pre-morbid level of functioning, were regular with medication and with follow up at the hospital. A little over half the subjects admitted that they believed that the disease model was their best explanation for their illness, while the rest preferred non-medical explanatory models.

**Table 1:** **Details of course andoutcome of the sampleat 5 years**

**Characteristic**	**No. (%)**	**Median**	**Mean (SD)**	**Range**
Remission	65 (68.4)	-	-	-
No. with relapses of psychosis after initial episode	24 (25.2)	-	-	-
No. who returned to pre-morbid functioning^1^	14 (14.7)	-	-	-
Regular hospital attendance over 5 years^2^	22 (23.2)	-	-	-
Regular medication compliance over 5 years^3^	24 (25.3)	-	-	-
Supervision of medication by family/carer^4^	43 (45.3)			
PANSS Total score	-	34	43.99 (17.42)	30-92
BPRS Total score	-	27	33.87 (15.85)	24-92
SAI-E Total score	-	33	26.02 (12.18)	1-35
WHODAS II Total score	-	8	12.72 (13.52)	0-48
Total duration of illness since onset (months)	-	80	91.3 (32.0)	24-252
Total duration in psychotic episodes since onset (months)	-	48	56.1(40.6)	8-192
No. of visits to hospital in 5 years^6^	-	19	22.3 (13.9)	2-54
No. of missed appointments over 5 years^6^	-	53	43.8 (23.4)	0-70
Preferred explanatory model at 5 year:		-	-	-
- disease model	52 (54.7)			
- non-medical model	43 (45.3)			

*BPRS*- Brief Psychiatric Rating Scale; *SAI-E*- Schedule for Assessment of Insight-Expanded; *PANSS*- Positive and Negative Syndrome Scale; *WHODAS-II*-WHO Disability Assessment Scale-II; 1-Pre-morbid functioning assessment by consensus between medical record and primary carer’s evaluation; 2- Regular hospital attendance over 5 years as per medical records; 3- Medication compliance assessment by consensus between medical record and primary carer's evaluation; 4- Supervision of medication by family/carer over 5-year period; 6- as per medical records.

### Correlations at 5^th^ year assessment

Insight scores at 5-year follow up was negatively correlated with psychopathology [BPRS total score (Pearson's Corr. coeff. -0.57; p = 0.000), PANSS total score (Pearson's Corr. coeff. -0.66; p = 0.000)] and disability (Pearson’s Corr. coeff. -0.53; p = 0.000) and positively with the total number of non-medical models (Pearson’s Corr. coeff. 0.37; p = 0.000). BPRS total score (β = −0.55; t = −6.13; p = 0.000), PANSS total score (β = −0.65; t = −0.77; p = 0.000), disability (β = −0.51; t = −05.6; p = 0.000) and the number of nonmedical explanatory models (β = 0.356 t = −0.32; p = 0.001) remained statistically significantly associated with insight after adjustment for age, sex, literacy and residence using multiple linear regression.

The total number of non-medical explanatory models at 5^th^ year follow up was significantly correlated negatively with psychopathology [BPRS score (Pearson’s Corr. coeff. -0.36; p = 0.000), PANSS total score (Pearson’s Corr. coeff. -0.42; p = 0.000)] and disability (Pearson’s Corr. coeff. -0.30; p = 0.003) and positively with insight scores (Pearson’s Corr. coeff.0.37; p = 0.000). BPRS total score (β = −0.32; t = −3.33; p = 0.001), PANSS total score (β = −0.39; t = −4.2; p = 0.000), disability (β = −0.26; t = −2.7; p = 0.000) and insight score (β = 0.31; t = −3.3; p = 0.001) remained statistically significantly associated with the total number of non-medical models after adjustment for age, sex, literacy and residence using multiple linear regression.

### Factors associated with clinical remission

Table 2 documents the factors associated with remission at 5 years on bivariate analysis. Table 3 and Table 4 record the relationships between remission on one hand and clinical variables and explanatory model variables respectively on the other, using multivariate procedures. Variables at baseline, which were statistically significant on bivariate analysis, and those variables significantly related at 0, 6, 12 and 60 months were employed for the multivariate statistical models. Clinical remission at 5 years was associated with pre-baseline factors (e.g. urban residence, fluctuating course of initial illness), improved functioning and shorter duration of total psychotic illness (e.g. better GAF scores at 6 months, lower psychopathology at 12 months). Insight scores and the presence of at least one non-medical explanatory model were only associated with remission later in the course of illness. The disease model was associated with remission while individual non-medical models (e.g. beliefs in evil spirit, black magic) were associated with poor outcome. These relationships remained statistically significant when logistic regression was used to adjust for factors associated with belief systems (e.g. age, sex, residence, education) and significant baseline variables. There was a significant interaction effect between disease explanation and non-medical explanatory models illness and remission. The relationship between remission and the different explanatory model variables, at 60 months, remained statistically significant even after adjusting for regularity of follow up over the 5-year period. Remission, insight scores and number of non-medical explanatory models of illness held were not significantly associated with number of hospital visits, the number of missed appointments or regularity of follow-up.

**Table 2:** **Factors associated with remissionat 5-year follow-up**

**Characteristic**	**Outcome status**	**Bivariate statistics**		
	**In remission No. (%)**	**Not in remission No.(%)**	**Odds ratio**	**95% Confidence interval**
Sex- male	38 (58.5)	11 (36.7)	2.43*	1.00- 5.93
Residence- urban	17 (26.2)	1 (3.3)	10.27**	1.30-81.30
No. of meals/day – 2 or less	22 (33.8)	4 (13.3)	3.33*	1.03-10.73
Duration of untreated psychosis >48 weeks^1^	27 (41.5)	19 (63.3)	0.41*	0.17-1.003
Fluctuating nature of initial illness	54 (83.1)	17 (56.7)	3.75**	1.42-9.91
Explanatory model- evil spirit	8 (12.3)	10 (33.3)	0.28*	0.10-0.81
SAI-E score >3^1^	41 (64.1)	13 (43.3)	2.33	0.96-5.65
**6-month data**				
SAI-E score >7^1^	33 (60.0)	7 (28.0)	3.86**	1.38-10.76
GAF score > 56^1^	33 (60.0)	7 (28.0)	3.86**	1.38-10.76
**12-month data**				
SAI-E score >14^1^	37 (61.7)	11 (37.9)	2.63*	1.06-6.56
BPRS score > 29^1^	26(43.3)	24 (82.8)	0.16***	0.05-0.47
GAF score > 64^1^	36 (60.0)	8 (27.6)	3.94**	1.50-10.33
Belief in disease model as causal for illness- present	30 (46.2)	5 (16.7)	4.29**	1.46-12.58
**60-month data**				
SAI-E score >33^1^	48 (73.8)	4 (13.3)	18.35***	5.59-60.28
BPRS score > 27^1^	1(1.5)	48 (73.8)	0.01***	0.002-0.097
WHODAS II score >8^1^	21 (32.3)	29(96.7)	0.017***	0.002-0.129
Belief in disease model as causal for illness- present	49 (75.4)	3 (10.0)	27.56***	7.37-103.1
Belief in evil spirits as causal for illness- present	1 (1.5)	4 (13.3)	0.10*	0.01-0.95
Belief in black magic as causal for illness- present	6 (9.2)	15 (50.0)	0.10***	0.03-0.31
At least one non-medical explanatory model	59 (90.8)	14 (46.7)	11.24***	3.72-33.91
Belief in disease model plus at least one non-medical model	48 (73.8)	3 (10.0)	25.41***	6.82-94.64
Preferred causal explanatory model - disease model	50 (76.9)	2 (6.7)	46.67***	9.94-219.04
Preferred causal explanatory model – non-medical model	15(23.1)	28 (93.3)	0.02***	0.005-0.10

Median used to dichotomize continuous variables; *BPRS*- Brief Psychiatric Rating Scale; *SAI-E*- Schedule for Assessment of Insight-Expanded; *PANSS*- Positive and Negative Syndrome Scale; OR- Odds Ratio; 95%CI- 95% Confidence Interval for Odds Ratio; * = p-value <0.05; ** = p-value <0.01; *** = p-value <0.001

The following variables were not statistically significantly related to outcome:

(i) at baseline- age, years of schooling, literacy, marital status, unemployment, unable to buy food, debt, requires carer, precipitating stress, visit to traditional healer, family history of mental illness, explanatory models related to disease, karma, punishment by God, black magic, involuntary admission status, BPRS scores, GAF score, at least one non- medical explanatory model held, 2 or more explanatory model held, disease model plus at least 1 non-medical model.

(ii) 6-months follow up data- BPRS scores, explanatory models related to disease, karma, punishment by God, black magic, evil spirit, at least one non- medical explanatory model held, 2 or more explanatory model held, disease model plus at least 1 non-medical model.

(iii) 12-month follow up data- explanatory models related to karma, punishment by God, black magic, at least one non- medical explanatory model held, 2 or more explanatory model held, disease model plus at least 1 non-medical model.

(iv) 60-months follow up data- karma, punishment by God, 2 or more explanatory model held.

(v) Over 5-year course- no. of hospital visits, no. of missed appointments, regularity of follow up.

**Table 3:** **Clinical characteristics associated withremission at 5-year follow-up-multivariate analysis**

**Model and Variables**	**Multivariate statistics**^**1**^	
	**OR**	**95% CI**
**Model for baseline data:**^2^		
Residence –urban	19.14	2.07-176.99
Fluctuating course of initial illness	5.16	1.66-16.02
**Model for variables at6-months:**^2^		
Fluctuating course of initial illness	2.54	1.03- 6.26
GAF score	2.74	1.20- 6.25
**Model for variables at12-months:**^2^		
Residence –urban	11.92	1.38-103.30
BPRS score	0.14	0.04-0.44
**Model for variables at60-months:**^2^		
BPRS score	0.008	0.000-0.17
SAI-E score	16.53	1.50-182.60
At least one non medical explanatory model present	18.69	1.60-218.95

*BPRS*- Brief Psychiatric Rating Scale; *SAI-E*- Schedule for Assessment of Insight-Expanded; *PANSS*- Positive and Negative Syndrome Scale; *OR*- Odds Ratio; 95%CI- 95% Confidence Interval for Odds Ratio; 1- logistic regression; 2- Statistically significant variables, on bivariate analysis, at specific point in time plus significant baseline variables (sex, residence, no. of meals/day, duration of untreated psychosis, fluctuating course of initial illness) included in the model.

**Table 4:** **Individual belief models andremission- multivariate analysis**

**Model and Variables**^**1**^	**Multivariate statistics**^**2**^	
	**OR**	**95% CI**
**Model for beliefs atbaseline:**^3^		
- Explanatory model- evil spirits as causal	0.18	0.04-0.75
- Residence –urban	34.07	2.96-391.95
-Fluctuating course of initial illness	4.18	1.30-13.43
**Model for beliefs at12-months:**^3^		
- Explanatory model- disease as causal	2.29	1.06-4.91
- Residence –urban	2.98	1.13-7.66
-Fluctuating course of initial illness	3.04	1.27-7.24
**Models for beliefs at60-months:**^3^		
I - Explanatory model- disease as causal	31.65	7.34-136.55
- Residence –urban	26.22	2.16-317.84
- Fluctuating course of initial illness	5.94	1.30-27.18
II- Explanatory model- black magic as causal	0.14	0.04-0.46
- Residence –urban	16.22	1.56-168.75
- Fluctuating course of initial illness	3.54	1.05-11.99
III- Preferred causal explanatory model – disease model	55.62	9.69-319.31
- Residence –urban	29.11	2.00-424.25
- Fluctuating course of initial illness	7.41	1.33-41.45
IV - Preferred causal explanatory model – non medical	0.02	0.003-0.10
- Residence –urban	29.14	2.00-424.25
V – Belief in at least one non-medical explanatory model	11.96	3.52-40.60
VI - Belief in disease model plus at least one non-medical model	61.45	16.27-232.04
- Residence –urban	3.63	1.03-12.77
VII - Interaction between disease and non-medical model	3.70	2.15-6.37
- Residence –urban	24.57	1.79-337.12

*OR*- Odds Ratio; 95%CI- 95% Confidence Interval for Odds Ratio; 1- No explanatory models significant at 6 months; 2- logistic regression; 3- model includes belief plus factors known to be associated with knowledge (age, sex, education, residence) and baseline clinical variables significantly associated with outcome (no. of meals/day, duration of untreated psychosis, fluctuating nature of initial illness).

The association between remission and changes in SAIE and BPRS scores between baseline and 6 and 12 months were tested for statistical significance. The only significant variable associated with remission were reduction in BPRS score between baseline and 12 months (t = −3.16; df = 87; p = 0.002), which remained significant (OR = 0.91; 95% CI 0.85-0.98; p = 0.01) after logistic regression analysis adjusting for other clinically significant variables. Changes in insight scores during the first year of treatment were not associated with outcome at 5 years.

### Analysis of repeated measures

**Figure 1:**
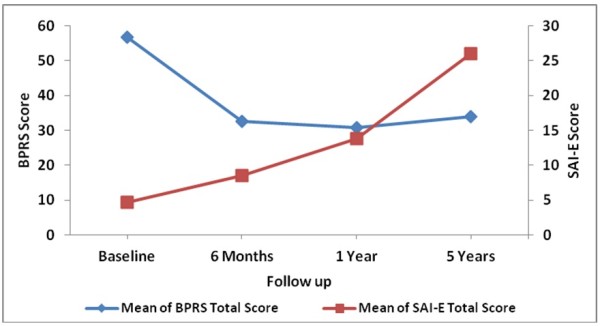
**Insight and psychosis ratingsover time.**

**Figure 2:**
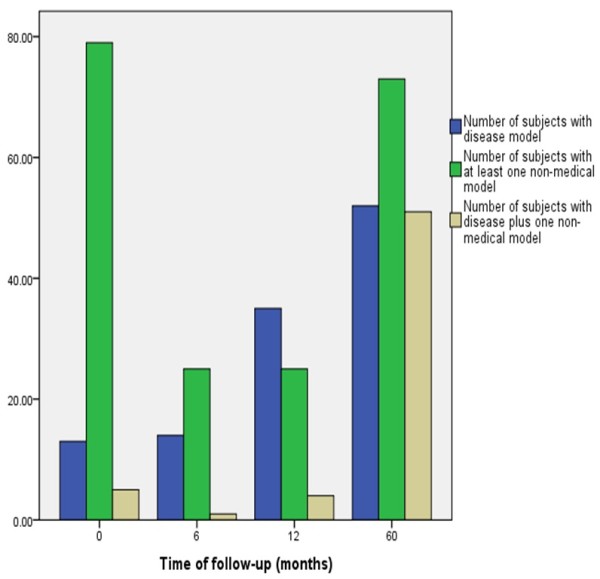
**Number and types ofexplanatory models over time.**

Figures 1 documents the serial insight and psychosis ratings and Figure 2 records the numbers of explanatory models of illness over the 5 year period. BPRS scores showed consistent decline while there was a steady increase in insight scores, number of patients who held disease model and those who simultaneously subscribed to disease and at least one non-medical model. The number of subjects who held at least one non-medical model was high at baseline, decreased with reduction in psychosis and treatment response and then seemed to increase with the stabilization of the course and outcome of illness.

The data was analysed using Generalized Estimating Equations (GEE) to assess the impact of serial assessments of SAI-E scores and the number of non-medical models on serial BPRS scores. Baseline variables significantly associated with remission and the number of visits to the hospital were entered as fixed variables in the model to adjust for their effects. Women (B = −2.65; SE = 1.03; p = 0.01), rural residence (B = − 2.06; SE = 1.00; p = 0.038), SAI-E scores (B = − 0.65; SE = 0.04; p = 0.000) and number of non-medical models (B = 5.31; SE = 0.91; p = 0.000) were significantly associated with BPRS scores. The non-significant variables in the model were: number of meals per day, duration of untreated psychosis, fluctuating initial course, and number of visits to hospital.

## Discussion

This is probably the first study with a large sample and a long period of follow-up, which has systematically examined psychopathology, insight, indigenous explanatory models of illness and outcome in schizophrenia. Its strengths include prospective cohort, detailed and repeated standard assessments of psychopathology and insight, assessment of both disease models and non-medical explanatory models of illness, a 5-year follow-up, and multivariate statistical procedures to adjust for confounders and to analyze repeated and correlated data. The fact that about a quarter of the original sample could not be examined at the 5^th^ year and the gap in psychopathology and insight assessments between 12 and 60 months are limitations.

The study has documented heterogeneity of outcome in schizophrenia. It recorded the simultaneous acceptance of multiple and contradictory explanatory models of illness and diverse sources of help seeking. Psychopathology, insight and explanatory models changed over the 5-year period. The proportion of patients holding disease models steadily increased over time with over half the subjects preferring it as the best explanation at 5 years. The number of patients holding non-medical models was high at recruitment, reduced over the first year of treatment and then rose dramatically. Urban residence, fluctuating course of the initial illness, improvement in functioning at 6 months and lower psychosis rating at 12 months were significantly related to remission. Insight scores and non-medical explanatory models held during the later course of the illness were significantly associated with outcome at 5 years. Disease models were associated with remission while non-medical were associated with poorer outcome. However, there was interaction between medical and non-medical models. The presence of the disease model and at least one indigenous model had a stronger impact on remission than the disease explanation per se. Analysis of longitudinal data showed that women, rural residence, insight scores and the number of non-medical explanatory models of illness held were significantly associated with BPRS scores during the study period.

### Insight and explanatory models

Many investigations have recorded the simultaneous presence of multiple and contradictory models of illness in people [8,18-23]. This is considered the norm in low and middle income countries but has also been recorded in western populations [20,24]. Previous studies have documented high insight scores in people who subscribe to disease models [8,9,19]. In addition, investigations who have examined non-medical models have demonstrated their negative relationship with insight scores [8,19]. Nevertheless, such correlations are natural considering the fact the instruments, which assess insight focus on disease explanations, attributions and actions, and concentrate on the recognition of mental illness attribution and seeking medical and psychiatric treatment. These instruments do not consider locally and culturally relevant attributions and help seeking.

### Insight, explanatory models and psychopathology

The reduction of psychotic symptoms and improved functioning during the first year of treatment is associated with an increase in the use of disease explanations and a marked reduction of causal non-medical beliefs. However, the number of people subscribing to disease explanations tends to plateau with only about half the subjects preferring it at 5 years. The pattern of change in the number of indigenous models of illness employed argues that they help coping. Their manifest reduction at 6- and 12-month follow-up coincides with the reduction in psychosis for all patients. Their marked increase at 60 months suggests their role as a coping strategy when the pattern of course and outcome of illness becomes established. Previous studies have documented the inverse relationship between psychopathology and insight [8,9,19,47-49]. However, many of these studies focused on cross-sectional and early illness data or had failed to adjust for many illness characteristics.

### Factors associated with outcome

The fact that pretreatment illness variables, improvement in functioning and reduction in psychopathology determined outcome at points in time before insight scores and explanatory models were significant suggest their major role in the process. The bivariate statistical association during the early course of illness between insight, explanatory models and outcome, lost their statistical significance when baseline and clinical variables were included in the multivariable analysis arguing that such relationships are confounded by illness characteristics. The independent predictor status of insight and explanatory models for clinical remission only becomes significant late in the course of illness. The positive relationship between insight and the number of non-medical explanatory models of illness, their negative relationship with psychopathology argues their impact on outcome is possibly secondary to the inherent nature of the disease. The relationships persisted after adjusting for the number of hospital visits.

These findings challenge the direction of the relationship between insight and psychopathology arguing that insight may be secondary to psychopathology. The association of insight and non-medical explanatory models to long-term outcome later in the course of illness suggests that explanatory models are coping mechanisms rather than being causally related to outcome.

The inverse correlation between insight scores and psychopathology in earlier reports [19,47-49] did not examine its impact on long-term outcome and take into consideration the time course of the illness. Although many studies have documented good insight as predictor of outcome, this relationship may be confounded by illness variables, which are usually not systematically excluded. The fact that pre-treatment variables and current psychopathology confound the relationship between explanatory models of illness at all points of time makes it difficult to conclusively argue that insight predicts longer-term outcome. Our data suggests the reverse, that psychopathology and illness characteristics predict insight, explanatory models and outcome in schizophrenia.

The heterogeneity of clinical features, course, outcome and treatment response of schizophrenia probably reflects different diseases, illnesses and trajectories. The fact that non-medical beliefs held during the later part of the illness were associated with non-remission and treatment non-response suggests that patients with a worse course and outcome and poorer response to treatment may select such explanatory models to cope with the devastating impact of their illness.

### Explanatory models, stigma and coping

Pluralistic societies employ multiple approaches to health and illness. The simultaneous use of contradictory models suggests their use in coping with different aspects of the condition [27]. The fact that people with non-medical beliefs were willing to take anti-psychotic medication argues for the complexity of the issues demanding a nuanced understanding of explanations and coping mechanisms. The persistence of the association between poor outcome and non-medical causal models, after adjusting for regularity of hospital follow-up, suggests that they are coping mechanisms for poor outcome conditions.

Mental illness and their labels are stigmatizing across cultures leading to much prejudice and discrimination. This is particularly significant in people with residual and poor outcome schizophrenia whose limited response to medication, adverse effects and continued symptoms demands the need for explanations, which go beyond simple disease. Non-medical, supernatural and external explanatory models seem to be preferred to disease explanations in order to cope with the devastation of the disease in people who have not recovered with treatment. They seem to be culturally acceptable mechanism to cope with complex and incapacitating outcomes. The persistence of multiple explanatory models of across cultures for chronic illnesses may suggest an evolutionary advantage.

### Insight as narrative

Medicine and psychiatry, despite their attempts at a universal understanding of mental disorders, cannot do justice to patients without the recognition of the contexts and the sharing of personal narratives. Psychiatry with its focus on objective behaviours, symptom checklists and diagnostic criteria has reduced the importance of patient experience and narratives [50]. Illness narratives contextualise the patient. They capture the individual’s suffering in an everyday context, in contrast to the medical narratives that reflect the needs of psychiatry. Patient experiences are particularly important for psychiatrists and mental health professionals who work every day with people with chronic and disabling mental illnesses but have never actually walked in their shoes. Illness narratives refocus the doctor-patient interaction and provide a window into the patient’s reality and his/her ways of coping [51].

The lack of laboratory tests to diagnose mental disorders tends to raise doubts about their diagnostic certainty making many patients and their families uncertain and uncomfortable about psychiatric labels [52]. In addition, negative attitude related to mental illness abound in most societies, result in stigma and lead to discrimination. The acceptance of such diagnostic labels can reduce self-esteem among patients. Patients, consequently, refuse to accept biomedical models and explanations for their experience and conditions.

The subjective dimension of insight has been conceptualized as a particular form of narrative production, called narrative insight [53]. Explanatory models of patients and their families are narratives to make sense of and convey experiences of illness, control them, maintain or improve quality of life [52]. These narratives can increase empathic understanding of illness experiences and life events, reduce their threat, provide some degree of control over these phenomena and incidents and improve their quality of life. They may often be effective aids to coping, particularly for treatment-resistant symptoms and deficits and incapacitating medication side effects.

There is a need to reconcile personal narratives with the biomedical model so that many personal strategies employed to coping with mental illness are not devalued or dismissed. Such experiential knowledge, although personal, will have to be recast in each case. It will help translate experience, provide legitimate frameworks and create a language and interface for improved communication [50]. The reframing of such narratives will impact psychiatric care, practice, research and health care delivery systems. Patient experience and perspectives, devalued and delegitimized by canonical authority, needs to be reemphasized and integrated into clinical practice. There is a need to foreground patient experience in order to impact the mainstream psychiatric discourse.

### Explaining insight

It has been argued that insight is shaped by psychology (i.e. motivation and denial) and constraints of biology (i.e. cognitive impairment and anosognosia) and is influenced by social constructions of illness (e.g. culturally specific explanatory models) [6,7]. Its comparison to anosognosia, as in brain disease and damage, may not be very useful as its response to antipsychotic medication and change over time and limit such comparison. Similarly, its attribution to cognitive impairment is less than definitive in view of its response to antipsychotic medication, which is generally not very useful in improving the many cognitive deficits in schizophrenia. Its reciprocal relationship with psychopathology during the initial course of schizophrenia suggests its association with delusional thinking and beliefs. Severe delusional illnesses, by their definition, preclude alternative explanations especially at the height of the illness and in those with severe disease.

The association of disease models of illness in those with good response to treatment and the correlation between non-medical illness explanations and poor response suggests their role in coping. The complex interaction between disease and indigenous models and the impact on remission argues for a nuanced understanding of the relationships between explanatory models and outcome. The complexity of schizophrenia, its heterogeneity in course and outcome, its varied response to antipsychotic medication and its unpredictable impact on diverse aspects of life seem to elicit a multifaceted response from people who suffer from it, their relatives and from society.

### Framing insight

Insight is not just the possession of discrete facts about the nature of mental illness. Nor is it only an acknowledgement of a particular experience as abnormal. It is also not an acceptance of a singular truth about the person and his/her life. An awareness of a mental illness is a narrative act in which people make personal sense of the many challenges they face. Such challenges include persistent psychotic symptoms, negative syndrome, cognitive deficits, impaired social relations, perpetual instability and livelihood issues. The patient’s narrative of the illness would then vary according complexity of the problem and context. The insight narrative would also vary according to its role in coping and adapting to the new challenges and demands. The cultural context, acceptable cultural explanations and the prevalent social stigma related to mental illness would interact with the person’s illness to produce a particular understanding. The complexity of issues calls for a nuanced framing of insight.

### Defining insight

Alternative conceptualisations of insight have employed findings from investigations in the non-western cultures [14,22-24,27,54]. These findings include: (i) patient’s often provide non-medical explanations for the cause of illness (e.g. karma, evil spirits, black magic, sin, punishment by God, etc.), (ii) mental illness labels are very stigmatising across cultures and settings, (iii) many subjects simultaneously hold multiple, diverse and contradictory beliefs and reflect the pluralistic approach of patients, (iv) many patients simultaneously seek biomedical and non-biomedical interventions from a variety of healers and centres of cure and healing. These findings argue that the current multidimensional models are not culturally sensitive to assess insight. They suggest that it is difficult to have definable, objective or universal standard for insight. They argues that there is a need for universal conventions to compare individual explanations with sub-cultural perceptions [14,54].

Alternative dimensions have been suggested for the assessment of insight (Table 5) [14,54]. Insight should be assessed against local and cultural standards of providing non-delusional and culturally acceptable explanations and attributions and seeking locally acceptable and available interventions. The findings of this study also suggest that subscribing to multiple explanatory models of illness argues that it is a pragmatic response to the devastation of chronic and residual psychosis. The assessment of insight should be similar to the assessment of other clinical phenomena like delusions, which involve comparison with local and cultural yardsticks [14,54].

**Table 5:** **Current and proposed dimensionsof insight**

**Current dimensions of insight**[4]	**Proposed dimensions of insight**[14,54]
Ability to re-label psychotic experience as abnormal	Ability to re-label psychotic experience as abnormal
Awareness of mental illness	Change is attributed to and corresponds with local beliefs for illness. This can include the simultaneous use of multiple and contradictory explanations.
Seek medical treatment	Acknowledge the need for restitution and seek available intervention/treatment. This can include the concurrent or sequential use of diverse interventions from varied facilities offering cure and healing^.^

The findings argue for the fact that the assessment of insight should be against the local cultural standards including disease models rather than universal yardsticks (e.g. of solely employing biomedical models). The assessment of insight should evaluate awareness, attribution and action. People with psychosis who are able to re-label their psychotic experience, offer non-delusional explanations for changes in themselves, which correspond to beliefs about illness held by the subculture, admit to the need for restitution, and seek locally available help, can be said to possess insight. The results recommend the use of universal conventions (of awareness, attribution to locally accepted non-delusional explanations and seeking available help) to assess insight in people with psychosis rather than the use of uniform criteria which focus solely on the biomedical model of disease.

### Clinical and research implications

Many people hold multiple causal and treatment models of their illness [55]. This is particularly true for chronic and disabling illnesses, which do not completely respond to medication and treatment. Attempts at elicitation of the locally relevant causal and treatment models of illness in addition to the biomedical model will provide clinicians with an understanding of residual problems and disability and the patient’s attempt at coping. While medication compliance is crucial to the reduction of psychotic symptoms and the maintenance of remission, other non-medical models also help in providing a pragmatic response to the persisting symptoms and problems. Non-medical beliefs should not be challenged or dismissed and the clinician should not claim exclusivity or superiority of the biomedical model. The use of diverse coping strategies for healing should be encouraged while emphasising medication compliance. Assessing insight in clinical practice will mandate the need to elicit all causal explanatory models and identify the diversity of help seeking. The use of a combination of different explanations including the biomedical model suggest attempts at coping employing many diverse culturally sanctioned strategies reflecting a greater awareness of the illness as well as the local context and environment (i.e. insight).

Research into insight in psychosis should also include non-medical belief models and move beyond pure biomedical explanations. There is a need to develop and refine instruments, which are able to capture diverse belief systems commonly employed by people with mental illness and also elicit local explanatory models of illness, their attribution and help seeking.

### Moving forward

The findings of this study mandate replication. The sole use of biomedical perspectives in the evaluation of response to complex diseases needs to be replaced with a broad based approach and understanding of coping across cultures. People employ diverse approaches to maintain mental health. The partial solutions offered by individual systems of medicine force people to employ diverse and multiple strategies to cope with distressing symptoms and intractable problems. Consequently, there is a need for a non-judgemental approach to the explanatory models employed by people with mental illness. While antipsychotic medication has a powerful impact on outcome, the use of other strategies especially in chronic conditions to cope with the variable impairment, disability and handicap, even among those with clinical remission, suggest the need to allow patients to use multiple strategies to regain and maintain mental health.

Cultural knowledge, empathy and an understanding of its complex interaction, which impact diseases and shape the illness response, is crucial to providing culturally sensitive care. Presenting biomedical perspectives without dismissing patient beliefs and negotiating a shared treatment plan without claiming exclusivity are cardinal. This will allow mental health professionals to bridge the disease-illness and the healing-cure divides across all cultures and settings. The complexity of the issues demonstrated highlight the need for a nuanced understanding of issues related to insight, explanatory models of illness and culture in schizophrenia and for other chronic illness.

## Conclusion

This 5-year cohort study of people with first episode schizophrenia systematically examined psychopathology, insight, explanatory models, course and outcome. Illness variables at baseline and during the early course of the illness predicted outcome at 5 years, while insight scores and non-medical explanatory models later in the course of illness were associated with long-term outcome. The positive relationship between insight and the number of non-medical explanatory models of illness, their negative relationship with psychopathology argues that association with outcome is possibly secondary to the inherent nature of the disease. These findings challenge the direction of the relationship between insight and psychopathology. They argue that insight and explanatory models are coping mechanisms secondary to psychopathology and course of the illness rather than being causally related to outcome.

Insight, the disease model and the number of non-medical model positively correlated with improvement in psychosis arguing for a complex interaction between the culture, context and illness variables. The awareness of mental illness is a narrative act in which people make personal sense of the many challenges they face. The course and outcome of the illness, cultural context, acceptable cultural explanations and the prevalent social stigma interact to produce a complex and multifaceted understanding of the issues. This complexity of issues related to disease, illness, context and culture call for a nuanced framing of insight.

## Competing interests

The authors do not have any competing interest to declare.

## Authors’ contributions

KSJ, SDM, HC and SJ designed the study. SJ collected the data. VJ and KSJ analysed the data. KSJ wrote the paper. All authors have read and approved the final draft.

## Pre-publication history

The pre-publication history for this paper can be accessed here:

http://www.biomedcentral.com/1471-244X/12/159/prepub
